# Friend or Foe? Recent Strategies to Target Myeloid Cells in Cancer

**DOI:** 10.3389/fcell.2020.00351

**Published:** 2020-05-19

**Authors:** Mehdi Chaib, Subhash C. Chauhan, Liza Makowski

**Affiliations:** ^1^Department of Pharmaceutical Sciences, College of Pharmacy, The University of Tennessee Health Science Center, Memphis, TN, United States; ^2^South Texas Center of Excellence in Cancer Research, School of Medicine, University of Texas Rio Grande Valley, Brownsville, TX, United States; ^3^Department of Immunology and Microbiology, School of Medicine, University of Texas Rio Grande Valley, Brownsville, TX, United States; ^4^Division of Hematology Oncology, Department of Medicine, The University of Tennessee Health Science Center, Memphis, TN, United States; ^5^Center for Cancer Research, The University of Tennessee Health Science Center, Memphis, TN, United States

**Keywords:** myeloid cells, Immunotherapy, MDSC, TAM, DC, TME, immune checkpoint blockade, microbiome

## Abstract

The tumor microenvironment (TME) is a complex network of epithelial and stromal cells, wherein stromal components provide support to tumor cells during all stages of tumorigenesis. Among these stromal cell populations are myeloid cells, which are comprised mainly of tumor-associated macrophages (TAM), dendritic cells (DC), myeloid-derived suppressor cells (MDSC), and tumor-associated neutrophils (TAN). Myeloid cells play a major role in tumor growth through nurturing cancer stem cells by providing growth factors and metabolites, increasing angiogenesis, as well as promoting immune evasion through the creation of an immune-suppressive microenvironment. Immunosuppression in the TME is achieved by preventing critical anti-tumor immune responses by natural killer and T cells within the primary tumor and in metastatic niches. Therapeutic success in targeting myeloid cells in malignancies may prove to be an effective strategy to overcome chemotherapy and immunotherapy limitations. Current therapeutic approaches to target myeloid cells in various cancers include inhibition of their recruitment, alteration of function, or functional re-education to an antitumor phenotype to overcome immunosuppression. In this review, we describe strategies to target TAMs and MDSCs, consisting of single agent therapies, nanoparticle-targeted approaches and combination therapies including chemotherapy and immunotherapy. We also summarize recent molecular targets that are specific to myeloid cell populations in the TME, while providing a critical review of the limitations of current strategies aimed at targeting a single subtype of the myeloid cell compartment. The goal of this review is to provide the reader with an understanding of the critical role of myeloid cells in the TME and current therapeutic approaches including ongoing or recently completed clinical trials.

## Introduction

Immune cell involvement in inflammatory ailments has long been established; however, their role in cancer remained unappreciated until the past three decades (Chen and Mellman, 2017). Indeed, the paradigm of cancer cells being a single player in cancer progression has shifted to models that include several stromal elements of the tumor microenvironment (TME) (Hanahan and Weinberg, 2011). The TME stroma is composed of endothelial cells, fibroblasts, extra cellular matrix, and diverse immune cell populations that act dynamically to regulate tumor growth. Myeloid cells play a major role in the body’s defense against infection, tissue homeostasis, as well as modulation of T cell mediated immunity (Merad et al., 2013; Cozzo et al., 2017). However, in the tumor, while myeloid cells initially respond to an injury or wound signal in the TME, neoantigen within the cancer cell, or some other signal from growing cancer cells, often the phenotype of immune cells evolve such that they become our own worst enemy in the fight against cancer.

Myeloid cells constitute a major stromal cell population in the TME (De Vlaeminck et al., 2016). They regulate tumor growth by direct or indirect interaction with cancer cells (Gabrilovich et al., 2012; Broz and Krummel, 2015). Myeloid cells comprise mononuclear and polymorphonuclear cells (Engblom et al., 2016). Macrophages are the major myeloid component of mononuclear phagocytes and represent the largest population of immune cell infiltrates in all tumors (Noy and Pollard, 2014), and as such are called “Tumor Associated Macrophages” (TAMs). TAMs are strongly linked to therapy resistance and are associated with poor prognosis (Kurahara et al., 2013) due to soluble factors secreted by infiltrating TAMs that contribute to drug resistance, metastasis, and immune evasion (Beatty et al., 2011). Macrophages are highly plastic cells capable of adopting different phenotypes in response to signals within various microenvironments (De Palma and Lewis, 2013). Thus, while TAMs have the capacity to kill cancer cells (Evans and Alexander, 1970), TAMs can also be modified to promote tumor growth and metastasis (Mantovani, 1978) - which emphasizes their critical and complex role in tumor biology.

Dendritic cells (DCs) are another mononuclear myeloid cell, although less prevalent, DCs are powerful components of the TME through their role as antigen presenting cells. Like TAMs, DCs have several phenotypes or subtypes which include classical DCs (cDCs) which are specialized in antigen presentation and induction of T cell immunity (Merad et al., 2013), plasmacytoid DCs (pDCs) which produce interferon-α which is important in antitumor immunity (Swiecki and Colonna, 2015), and monocytic DCs (mDCs) which differentiate from circulating monocytes and present a pro-inflammatory phenotype (Gopalakrishnan et al., 2018). Another family of myeloid cells are myeloid-derived suppressor cells (MDSCs), which are potent immunosuppressive cells that arise in pathological conditions such as cancer. MDSCs promote tumor growth, angiogenesis and metastasis, but their main function is to suppress T cell activation leading ultimately to immune evasion (Talmadge and Gabrilovich, 2013). MDSCs are an immature abnormally differentiated class of myeloid cells which comprise two distinct classes: granulocytic or polymorphonuclear MDSC (PMN-MDSC) and monocytic MDSC (M-MDSC) (Marvel and Gabrilovich, 2015). Interestingly, M-MDSCs have been shown to differentiate into TAMs in tumors, suggesting that targeting only one subtype of tumor infiltrating myeloid cell such as PMN-MDSCs may not be sufficient to achieve an effective therapeutic response (Kumar et al., 2016). In this review, we define the role of myeloid cells in cancer, with a focus on TAMs and MDSCs, and how they contribute to immune suppression and therapy resistance. We also summarize novel molecular targets in myeloid cells and discuss up-to-date strategies, such as targeted delivery, to effectively deplete or reconvert our foes to friends in the TME to increase therapeutic efficacies to best fight cancer. Our overall goal is to convey to our readers the importance of targeting myeloid cells in cancer, while critically emphasizing the limitations of current monotherapies targeting myeloid cells in malignancies.

## Myeloid Cell Phenotypes in Cancer

Cancer cells exploit myeloid cells to escape immune surveillance by changing their phenotype from tumoricidal to tumor supportive and immunosuppressive (Awad et al., 2018). Myeloid cells play an important role in tissue homeostasis and regulation of adaptive immune responses by regulating CD4 and CD8 T cell content and activation. Thus, myeloid cells are highly versatile and plastic cells making them suitable pharmacologic targets to attempt to revert their phenotype to overcome immune tolerance in cancer (Schouppe et al., 2012).

### Macrophages

Macrophages are plastic cells of the innate immune system capable of adopting varied phenotypes in response to signals in their microenvironment (Okabe and Medzhitov, 2014). In pathological conditions, macrophages respond to pathogen-associated molecular patterns (PAMPs) like lipopolysaccharide (LPS) derived from gram negative bacteria, which then activate transcription factors such as nuclear factor kappaB (NF-kB) through toll-like receptor 4 (TLR4) to initiate an inflammatory response (Kawai and Akira, 2010). Pro-inflammatory (M1-like) macrophages secrete cytokines such as IL-12, IL-6, and TNF-α to amplify the pro-inflammatory response against pathogens by recruiting more leukocytes to the site of inflammation (Ngambenjawong et al., 2017). In contrast, alternatively activated macrophages (M2-like) are present in wound healing environments in response to IL-4 and IL-13 cytokines. This stimulation results in the production of anti-inflammatory enzymes such as arginase (Arg-1) in a STAT6-dependent manner, producing a cascade of immunoregulatory and tissue remodeling events through the secretion of key cytokines and metabolites by alternatively activated macrophages (Dyken and Locksley, 2013). Similarly, in the TME, M2-like macrophages produce cytokines, chemokines, and enzymes that have tumor promoting properties (Castells et al., 2012). Recent evidence suggests that most tissue-resident macrophages arise from fetal precursors in the yolk sac independently of bone marrow-derived cells and persist throughout life (Ginhoux and Guilliams, 2016). Yet the origin of TAMs is complex and dependent upon the tumor milieu (Wynn et al., 2013). In breast, lung, pancreas, brain, and liver mouse cancer models, tissue resident-derived TAMs are progressively diluted by monocyte-derived TAMs (mo-TAMs) during tumor growth (Lahmar et al., 2016). For example, TAMs in the MMTV-PyMT mammary tumor model are phenotypically and functionally distinct from tissue-resident macrophages and are derived from circulating monocytes (Franklin et al., 2014). In contrast, a significant portion of pancreas-resident macrophages originate from embryonic development in Pancreatic Ductal Adenocarcinoma (PDAC) mouse models (Zhu Y. et al., 2017). Despite the controversy regarding the origin of TAMs and complexity of cancer specificity, together the evidence suggests that the ontogeny of TAMs is heterogenous and that both monocyte-derived and tissue resident macrophages constitute the pool of TAMs that infiltrate primary and metastatic tumors (De Palma, 2016). For example, in *Ccr2-/-* mice engrafted with colorectal cancer, reduction in monocyte-derived TAMs was associated with reduced tumor burden suggesting a role of mo-TAMs in tumor growth (Afik et al., 2016). Although monocyte-derived TAMs and tissue resident TAMs play different roles during tumor progression, as previously reported in PDAC and brain cancer mouse models (De Palma, 2016; Zhu Y. et al., 2017), more evidence is needed to accurately define the contribution of varied TAM subpopulations to more efficient targeting in malignancies.

Clinically, high densities of macrophages in primary tumors have been correlated with poor prognosis (Mantovani et al., 2017). However, both positive and negative outcomes have been reported in colon, lung, prostate, and bone cancers in the presence of high TAM content (Zhang et al., 2015). It is possible that these conflicting data are related to the type and stage of cancer or to the type of analysis performed (Ruffell and Coussens, 2015). The presence of the M1-like phenotype in TME correlates with a better prognosis, while the presence of the M2-like phenotype usually predicts poorer prognosis (Yuan et al., 2014). TAMs were also reported to mediate chemotherapy resistance in various cancer types by activating anti-apoptotic pathways and/or by providing cancer cells with survival factors (Ruffell and Coussens, 2015). While detailed causes of TAM-induced tumor growth and therapy resistance have yet to be uncovered, emerging therapeutic approaches aiming to deplete macrophages and/or shift macrophage phenotypes represent promising therapeutic modalities for cancer patients (Quail and Joyce, 2017).

### Myeloid-Derived Suppressor Cells (MDSCs)

Myeloid-Derived Suppressor Cells are only found in pathologic conditions such as cancer, obesity, autoimmunity, or chronic infection. In contrast to most other myeloid cells, MDSCs are strongly immunosuppressive. In cancer, MDSCs are derived from myeloid progenitor cells and accumulate in the bone marrow in response to signals released by tumors (Condamine et al., 2015a). Activation of MDSCs results from a continuous stimulation of myeloid cells with low-strength signals, causing poor phagocytic capacity, and elevated production of reactive oxygen species (ROS), nitric oxide (NO), and anti-inflammatory cytokines (Kumar et al., 2016). The abundance of tumor infiltrating MDSCs is associated with advanced malignancy stage and an overall poorer prognosis in various types of cancer (Parker et al., 2015). For example, patients with stages III and IV melanoma, non-small cell lung cancer, hepatocellular carcinoma, pancreatic, bladder, and gastric cancers have higher frequencies of circulating MDSC in the peripheral blood as compared to patients with stages I and II of these diseases (Almand et al., 2001; Gabitass et al., 2011; Eruslanov et al., 2012; Jiang et al., 2015). Additionally, solid tumor patients who have high levels of circulating MDSCs respond poorly to immunotherapy such as immune checkpoint inhibitors (Weber et al., 2018). There are two types of MDSCs that have been identified in both mice and humans: polymorphonuclear MDSCs (PMN-MDSC) that are morphologically similar to neutrophils, and monocytic MDSCs (M-MDSC) that are similar to monocytes (Condamine et al., 2015b; Ugel et al., 2015). A third class of MDSCs was recently described in human peripheral blood mononuclear cell (PBMC) and is referred to as “early-stage MDSC” (eMDSC). eMDSCs lack the expression of CD14 which is expressed in human M-MDSC and CD15 which is expressed in human PMN-MDSC. However, eMDSC specific role and its mouse equivalent population are yet to be defined (Bronte et al., 2016). MDSCs are functionally defined by their ability to suppress antitumor T cell activity through the secretion or expression of immune-regulatory factors including Arg1, NO, TGF-β, and cyclooxygenase 2 (Vasquez-Dunddel et al., 2013; Marvel and Gabrilovich, 2015). For example, Arg1 depletes arginine which is an essential amino acid for T cell proliferation and activation, while reactive oxygen species produced by MDSCs kills target cells by inducing oxidative stress (Ostrand-Rosenberg and Fenselau, 2018). PMN-MDSCs are recruited to the tumor site primarily by the CXC chemokine family which include CXCL1, 5, 6, 8, and 12 (Kumar et al., 2016). In a mouse model of hepatocellular carcinoma, increased production of CXCL12 promoted CXCR4-mediated recruitment of PMN-MDSCs to premetastatic niche sites (Seubert et al., 2015). Similarly, loss of CXCR2 in a colitis-associated cancer mouse model dramatically inhibited tumorigenesis through inhibiting infiltration of PMN-MDSCs into colonic mucosa and the tumor site (Katoh et al., 2013). In contrast, M-MDSCs are recruited to primary and metastatic tumor sites through chemokines produced by tumors, primarily CCL2 and CCL5 (Kitamura et al., 2015; Kumar et al., 2016). Clinically, MDSCs have been suggested as predictive biomarkers for disease outcome as high levels of circulating MDSCs prior to cancer therapy negatively influenced survival in most cancers suggesting that circulating MDSCs should be taken into account to improve prognostic evaluation (Wang P.F. et al., 2018). Taken together, these studies demonstrate the need for an effective targeting of MDSCs in cancer to overcome limitations of current treatment options such as chemotherapy and immunotherapy.

### Other Myeloid Cell Subtypes

**FIGURE 1:**
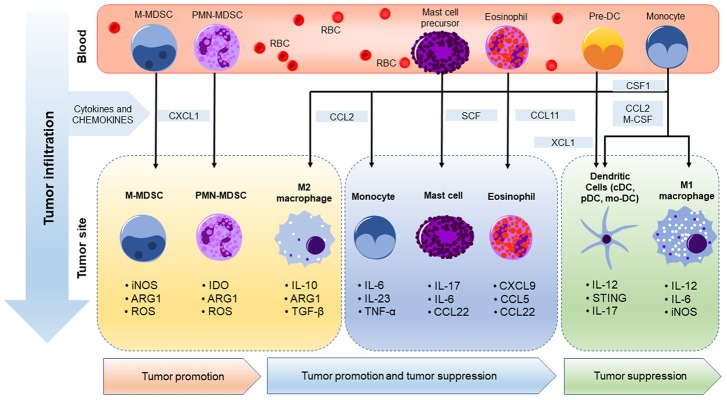
Myeloid cell phenotypes in cancer. In cancer, myeloid cells are generated in the bone marrow from the common myeloid progenitors and migrate to the tumor microenvironment in response to factors released by tumors. Suppressor myeloid cells such as MDSCs and M2 macrophages promote tumor growth by suppressing innate and adaptive immunities via production of immune suppressive factors such as arginase 1 and other cytokines. These factors promote an immunosuppressive tumor microenvironment by altering innate anticancer immunity and T cell functions. On the other hand, M1 macrophages and DCs have antitumor potential via production of pro-inflammatory and antitumor factors such as IL-12 and iNOS. While the role of macrophages, MDSCs, and DCs are best studied and are the main focus of this review, the role of monocytes, and less prevalence (lower density cells) mast cells and eosinophils in tumor promotion and suppression is less clear and is an active area of research while further evidence is required to fully elucidate their role in cancer immunity (Jachetti et al., 2018; Rigoni et al., 2018; Gorzalczany and Sagi-Eisenberg, 2019).

Dendritic cells are versatile antigen-presenting cells which have the ability to initiate pro-inflammatory immune responses and are major contributors to cytotoxic responses in tumors (Worbs et al., 2016). Conventional DCs (cDCs), among other DC subtypes, preferentially activate T cells which represent the foundation of the “cancer-immunity cycle” (Chen and Mellman, 2013). cDCs can be divided into two different subsets: cDC1 and cDC2 (Merad et al., 2013; Guilliams et al., 2014). cDC1 depend on the transcription factors IRF8, Batf3, and ID2 for development and express CD103 in mice while CD141 is used to distinguish cDC1 in humans (Böttcher and Reis e Sousa, 2018; Collin and Bigley, 2018). cDC1 are essential for CD8+ T cell activation as highlighted by several studies using cDC1-deficient *Batf3*^–/–^ mice and other *in vivo* models of cDC1 depletion, which consistently display a loss of cDC1’s ability to induce a T cell-mediated antitumor immune response (Broz et al., 2014; Salmon et al., 2016; Sanchez-Paulete et al., 2016; Spranger et al., 2017). cDCs activate CD8+ T cells by cross-presenting extracellular antigens on major histocompatibility complex class molecules (Broz and Krummel, 2015). Hence, high numbers of tumor infiltrating DCs were associated with T cell activation resulting in an antitumor immune response (Dieu-Nosjean et al., 2008; Goc et al., 2014). Additionally, the presence of cDC1 in the tumor stroma was correlated with increased overall survival in patients with various types of cancer (Broz et al., 2014). However, tumors may also alter the anti-cancer role of DCs (Gabrilovich, 2004) through several factors present in the TME. For example, IL-10 produced by TAMs prevents the production of IL-12 by CD103+ DCs leading to the impairment of T cell activation. Also, tumor microenvironmental factors such as low pH, hypoxia, and lactic acid impair DC-mediated T cell activation (Veglia and Gabrilovich, 2017). Clearly, like so many immune cells in the TME, the specific cancer, stage, aggressiveness, and other factors influence the phenotype of DCs. Finally, just like macrophages and DCs, neutrophils, eosinophils, mast cells, and monocytes have all been reported to adopt different phenotypes in cancer (Engblom et al., 2016; Porrello et al., 2018). Thus, myeloid cells represent a suitable and a major target in cancer despite the challenges they pose in our ability to distinguish their tumor-promoting versus inhibitory activities in preclinical models and cancer patients (Engblom et al., 2016; Porrello et al., 2018). The major tumor-infiltrating myeloid cell phenotypes in cancer are summarized in Figure 1.

## Novel Molecular Targets in Tumor Associated Myeloid Cells

Given the critical role that myeloid cells play in cancer, the need to identify novel molecular targets to block the recruitment of myeloid cells to the tumor site, shift their phenotype to an anticancer one, or simply deplete them may be of the utmost importance. Here we summarize recent findings of novel key players that modulate myeloid cell phenotypes in malignancies.

### PI3Kγ

The phosphoinositide 3-kinase (PI3K)-AKT-mTOR pathway controls key cellular processes such as growth, proliferation, and metabolism in cancer cells and is one of the most dysregulated pathways in malignancies (Thorpe et al., 2015; Janku et al., 2018). The class I PI3K lipid kinases drive metabolic and transcriptional pathways in inflammation and cancer (Martini et al., 2014). The PI3K Class 1A isoforms include PI3Kα and PI3Kβ which are widely expressed in epithelial and endothelial cells, while the Class IA isoform PI3Kδ is expressed mainly in lymphocytes. Importantly, the class 1B isoform PI3Kγ has a unique structure and is largely expressed in myeloid cells. PI3Kγ plays a major role in myeloid cell migration and accumulation in tumor tissues (Schmid et al., 2013; Martini et al., 2014). Recent studies have reported that PI3Kγ constitutes a molecular switch which controls macrophage polarization during inflammation and cancer (Kaneda et al., 2016b). PI3Kγ promoted immune suppression in malignancies through activation of Akt and mTOR signaling and prevention of NF-κB activation. Selective inactivation of PI3Kγ with the specific inhibitor IPI-549 (Evans et al., 2016) stimulated and prolonged NF-κB activation, thus alleviating immune suppression and restored CD8+ T cell cytotoxicity (Kaneda et al., 2016b). In two PDAC mouse models, pharmacologic blockade of PI3Kγ with the selective inhibitor TG100-115 reprogrammed TAMs to stimulate CD8+ T cell-mediated tumor suppression and inhibited tumor cell metastasis and desmoplasia, a fibrotic phenotype associated with TAMs and poor therapeutic efficacy (Kaneda et al., 2016a). Also, genetic or pharmacological inhibition of PI3Kγ or its downstream signaling molecule integrin α4 blocked MDSC recruitment to tumors and immune suppressive myeloid cell polarization, thus increasing expression of pro-inflammatory cytokines and reducing expression of anti-inflammatory cytokines. Moreover, inhibition of either PI3Kγ or integrin α4 stimulated DC and CD8+ T cell recruitment to the tumor site, thereby promoting tumor cell cytotoxicity (Foubert et al., 2017). In sum, targeting myeloid cell PI3Kγ in cancer patients can enhance the efficacy of current therapy regimens and may constitute a novel approach to improve the long-term survival of cancer patients (Gunderson et al., 2016; Kaneda et al., 2016b).

### PD-1- PD-L1 Axis

The programmed cell death protein 1 (PD-1) and programmed cell death ligand 1 (PD-L1) pathway are key components of the immunosuppressive TME (Rosenblatt and Avigan, 2017). PD-L1 is expressed on a variety of cell types including mesenchymal cells such as adipocytes (Wu et al., 2018), and is also found on hematopoietic cells such as lymphocytes and myeloid cells (Sharpe and Pauken, 2017). PD-1 is expressed during T cell activation and engages its ligands PD-L1 and PD-L2, thus inhibiting effective T cell cytotoxicity resulting in poor anti-tumor immunity (Greenwald et al., 2005; Sun et al., 2018). PD-L1 is expressed on TAMs, and the response rate to anti-PD-L1 antibody in patients where at least 10% of macrophages express PD-L1 was as high as 80% (Herbst et al., 2014). PD-L1 is also expressed on DCs, MDSCs, and monocytes (Sipe et al., 2020). *In vitro* treatment of macrophages with anti-PD-L1 antibody led to the activation of multiple macrophage pro-inflammatory pathways (Hartley et al., 2018). Additionally, combined treatment of anti-PD-1 and anti-PD-L1 antibodies cured half of the treated mice in an established melanoma mouse model (Hartley et al., 2018). These findings suggest that PD-L1 induces an immune-suppressive macrophage phenotype while treatment with anti-PD-L1 antibody reverses macrophage polarization, thereby triggering a potent macrophage-mediated anti-tumor immune response (Hartley et al., 2018). In another study, depletion of myeloid cells in a *Kras*-driven pancreatic cancer mouse model prevented tumor initiation and, in some cases, arrested tumor growth by restoring CD8+ T cell anti-tumor immunity. These results suggest that myeloid cells inhibit CD8+ T cell antitumor capacity by inducing PD-L1 expression in tumor cells (Zhang et al., 2017). Interestingly, PD-1 expression is not restricted to lymphocytes, but is also expressed by TAMs (Gordon et al., 2017). PD-1 expression in TAMs correlated positively with disease stage in both mice and humans with primary cancers. PD-1 expression by TAMs prevented phagocytosis of tumor cells by macrophages, whereas blockade of the PD-1-PD-L1 pathway *in vivo* restored macrophage phagocytic potential. These data suggest that PD-1 and PD-L1 blockade may also directly act on macrophages (Gordon et al., 2017). Consequently, myeloid cells represent an additional highly prevalent and potentially potent target for immune checkpoint blockade therapies in cancer.

### Iron Metabolism

Iron is a vital nutrient that enables cell proliferation and growth (Torti and Torti, 2013). It is required for oxygen transport, DNA biosynthesis, and the production of adenosine triphosphate (ATP) via electron exchange (Kosman, 2010; Soares and Hamza, 2016). There is ample evidence that iron overload is associated with cancer (Torti and Torti, 2013; Manz et al., 2016). Macrophages play a major role in iron homeostasis by recycling iron from senescent and dying red blood cells (RBC) back into the circulation and different tissues in the body (Andrews, 1999; Ganz, 2012; Hubler et al., 2015). Additionally, macrophage polarization is closely associated with iron metabolism (Dong et al., 2019). Studies have shown that over 60% of iron metabolism-related genes are differentially expressed between the M1/M2 macrophage axis (Recalcati et al., 2010). It is now established that M1-like macrophages express high levels of iron storage protein ferritin and low levels of iron export protein ferroportin (*Fpn*) which favor an iron-sequestration macrophage phenotype. On the other hand, M2-like macrophages display the iron-export phenotype by increased expression of ferroportin and decreased expression of ferritin (Recalcati et al., 2010; Jung et al., 2015). Consistent with these findings, higher iron availability in the TME was linked with accelerated ferroportin-mediated iron release by TAMs, which further validates the pro-tumorigenic properties of TAMs (Biswas and Mantovani, 2010; Marques et al., 2014). Moreover, iron-export TAMs express high levels of CD163, a high-affinity scavenger receptor for haptoglobin that also binds to hemoglobin. Activation of CD163 by binding to either hemoglobin or haptoglobin induces the transcription of ferroportin (Marro et al., 2010; Gnerlich et al., 2011). Previous evidence suggested that a high density of CD163+ TAMs positively correlated with poor prognosis in many cancer types (Heusinkveld and van der Burg, 2011). A recent study revealed that CD163+ TAMs promoted expression of IL-6 and CXCL2 by cancer cells while inhibition of either IL-6 or CD163 macrophage-induced tumorigenesis in a co-culture *in vitro* system and a sarcoma mouse model (Shiraishi et al., 2018). In addition, a study has shown that inhibition of heme oxygenase 1 (HO-1; which is an enzyme that degrades heme to release iron) in TAMs induced M1 polarization in macrophages and reduced tumor growth in a breast cancer mouse model (Mertens et al., 2016). Remarkably, the macrophage iron phenotype becomes a suitable target for iron chelators and iron oxide nanoparticles (IONs). Iron chelators have been explored as a monotherapy, or as an adjuvant therapy for the treatment of various malignancies (Heath et al., 2013). IONs on the other hand, have been broadly explored in preclinical and clinical studies in the past decade (Hu et al., 2018). IONs accumulation in macrophages increased intracellular iron levels, thus promoting the proinflammatory phenotype (Laskar et al., 2013; Dong et al., 2019). Ferumoxytol, an FDA-approved ION for the treatment of anemia, inhibited tumor growth and metastatic spread in a xenograft mouse model by shifting TAM polarization toward the M1-like phenotype (Zanganeh et al., 2016). Consequently, modulation of iron metabolism in macrophages may represent a promising monotherapy, or combination therapeutic approach for cancer patients.

### Microbiome

In recent years, the microbiome has emerged as a contributor of cancer progression in a variety of malignancies including colon and primary liver cancers (Plottel and Blaser, 2011). A recent study found that the malignant pancreas comprises a more abundant microbiome than the normal pancreas in both mice and humans (Pushalkar et al., 2018). Bacterial ablation in PDAC bearing mice reduced MDSC populations, increased M1 macrophage populations, promoted differentiation of CD4+ T cells, and activated CD8+ T cells to reduce tumor growth. These data suggest that endogenous microbiota promote immune suppression in PDAC patients and propose the microbiome as a potential target for the modulation of PDAC progression (Pushalkar et al., 2018). Another recent study has shown that *Peptostreptococcus anaerobius* which is an anaerobic bacterium, adheres to colorectal cancer cell mucosa and accelerates colorectal cancer development. Mechanistically, a *Peptostreptococcus anaerobius* surface protein, putative cell wall binding repeat 2 (PCWBR2) interacts with α_2_/β_1_ integrin in colon cancer cells which leads to the activation of the PI3K-Akt pathway resulting in NF-κB activation. NF-κB in turn triggers a pro-inflammatory response and leads to a significant expansion of MDSCs and TAMs. Pharmacological blockade of integrin α_2_/β_1_ impairs *Peptostreptococcus anaerobius* attachment and decreases tumor burden. These findings propose *Peptostreptococcus anaerobius*-induced PCWBR2-integrin α_2_/β_1_ axis as a potential therapeutic target in colorectal cancer (Long et al., 2019). The adaptor protein Caspase Recruitment Domain-containing protein 9 (CARD9) is exclusively expressed in myeloid cells and is required for the activation of innate immunity (Jia et al., 2014). Recent evidence suggests that CARD9 deficiency impaired macrophage fungicidal functions which led to increased fungal loads and a notable increase in *Candida tropicalis* in a colorectal cancer mouse model (Wang T. et al., 2018). *C. tropicalis* expansion induced accumulation of MDSCs which promoted tumor growth. Treatment of CARD9 deficient tumor-bearing mice with an anti-fungal fluconazole suppressed tumor growth in the colorectal cancer mouse model (Wang T. et al., 2018). These findings suggest a direct role of the microbiome in generating MDSCs and that targeting certain fungal populations within the microbiome may represent an attractive therapeutic approach in patients with colorectal cancer. In addition, macrophage-secreted human cationic antimicrobial protein 18 leucine leucine-37 (hCAP-18/LL-37) increased pancreatic cancer stem cell (CSC) pluripotency genes, self-renewal, and tumorigenicity. hCAP-18/LL-37 is an antimicrobial peptide secreted by activated macrophages, but its tumorigenic properties were previously unknown. Indeed, pharmacological inhibition of formyl peptide receptor 2 (FPR2) and/or P2X purinoceptor 7 receptor (P2X7R) on CSCs which are the receptors of hCAP-18/LL-37 inhibited tumor formation in a PDAC mouse model. Thus, hCAP-18/LL-37 is a novel, previously unrecognized target in TAMs to overcome CSC-induced relapse in cancer patients and an excellent example of microbially-mediated modulation of cancer progression.

### CXCR2

CXCR2 is a G-protein coupled receptor of the CXC chemokine family which is predominantly expressed on neutrophils and MDSCs (Dart, 2016). The primary immune function of CXCR2 is the regulation of neutrophil and MDSC migration and recruitment to inflammation including tumor sites (Cacalano et al., 1994; Eash et al., 2010; Bian et al., 2014; Highfill et al., 2014). Previous studies have reported that CXCR2 promotes tumorigenesis in skin and colon cancers (Jamieson et al., 2012). In a PDAC mouse model, genetic ablation of CXCR2 abrogated metastasis while pharmacological inhibition of CXCR2 decreased tumor growth. Inhibition of CXCR2 altered neutrophil/MDSC recruitment and enhanced T cell infiltration into the tumor site (Steele et al., 2016). In a breast cancer mouse model, CXCR2+ MDSCs promoted tumor growth and metastasis by secretion of IL-6 and modulation of CD4+ and CD8+ T cell recruitment to the tumor site. CXCR2+ MDSCs also upregulated the expression of inhibitory immune checkpoints PD-1, PD-L1, and cytotoxic T lymphocyte antigen 4 (CTLA4) as well as lymphocyte activation gene protein 3 (LAG3) on CD4+ and CD8+ T cells promoting immunosuppression (Zhu H. et al., 2017). Together, these findings propose CXCR2 as a suitable target to alleviate myeloid cell-induced immune suppression for a better therapeutic outcome in cancer patients.

## Specific Novel Molecular Targets in Myeloid Cells

Herein, we summarize the major novel molecular targets of myeloid cells in cancer from recent literature. Previous reviews have discussed other molecular targets in more detail which may be of interest (Noy and Pollard, 2014; Engblom et al., 2016; Mantovani et al., 2017).

### Molecular Targets in TAMs

Proteins secreted from or present in TAMs that have been recently published include mediators of fibrosis, inflammatory signaling, phagocytic capacity, lipid metabolism, and growth factors. First, TAMs in PDAC secrete granulin that activates resident hepatic stellate cells, which then secrete periostin, resulting in a fibrotic TME that promotes metastatic tumor growth. Alteration of TAM recruitment or granulin secretion abrogated liver metastasis (Nielsen et al., 2016). In a follow-up study, macrophage-derived granulin expression was induced in response to CSF-1 and caused CD8+ T cell exclusion in metastatic livers. Interestingly, genetic depletion of granulin diminished the establishment of a fibrotic stroma, thus restoring T cell infiltration at the metastatic site. Furthermore, depletion of granulin sensitized PDAC tumors to anti-PD-1 therapy, and dramatically reduced metastasis, suggesting that targeting TAM-derived granulin may sensitize PDAC tumors to immune checkpoint blockade therapies (Schmid et al., 2018). Second, TAM NOD-like receptor family pyrin domain-containing 3 (*NLRP3*) signaling promoted CD4+ T cell differentiation into regulatory T cell populations while inhibiting CD8+ T cell activation in an IL-10-dependent manner in a PDAC mouse model. Genetic or pharmacological inhibition of the NLRP3 complex resulted in the restoration of innate and adaptive anti-tumor immune response, suggesting that NLRP3 may represent a suitable target to sensitize PDAC to immunotherapy (Daley et al., 2017). Third, neuropilin-2 (NRP2), which is a member of the membrane-associated neuropilin family, is expressed during macrophage differentiation and is induced by tumor cells (Dai et al., 2017; Miyauchi et al., 2018). NRP2 in TAMs induced efferocytosis, which is the phagocytic clearance of dying cells, and promoted tumorigenesis because efferocytosis induces an M2-like anti-immune phenotype in macrophages (Morioka et al., 2018). Inhibition of NRP2 in TAMs increased secondary necrosis by impairing the clearance of dying cancer cells and promoted CD8+ T cell and natural killer (NK) cells infiltration (Roy et al., 2018). Fourth, caspase-1 was reported to promote TAM differentiation by cleaving peroxisome proliferator-activated receptor gamma (PPARγ) (Niu et al., 2017). PPARγ fragments then interacted with and attenuated medium-chain acyl-CoA dehydrogenase (MCAD) resulting in the promotion of TAM differentiation. Caspase-1 inhibition substantially inhibited tumor growth, thus proposing the caspase-1/PPARγ/MCAD pathway as a promising target to prevent TAM-induced tumorigenesis (Niu et al., 2017). Fifth, in BRAF-mutant models, BRAF inhibitors activated the mitogen-activated protein kinase (MAPK) pathway in macrophages which then produced VEGF to promote melanoma tumor growth. Macrophage-mediated resistance to BRAF inhibitors in melanoma was then reversed by blocking the MAPK pathway or macrophage-secreted VEGF. These results suggest that targeting TAMs may benefit BRAF-mutant melanoma patients (Wang et al., 2015). Sixth, ornithine decarboxylase (ODC) is an enzyme that limits polyamine biosynthesis. Consistent with previous literature reporting that ODC reduces M1 polarization in infection sites, a new study revealed that macrophage ODC also impaired the M1 phenotype and promoted colitis-associated colon carcinogenesis (Singh et al., 2018). Mice lacking ODC in myeloid cells demonstrated improved disease outcomes, suggesting that macrophage ODC is a suitable target for colon cancer chemoprevention (Singh et al., 2018). Finally, migration of TAMs is an exciting pathway to target. TAM mesenchymal migration is protease-dependent in mouse and human tumors, providing a new strategy for macrophage immunotherapy by targeting TAM motility (Gui et al., 2018). TAMs secrete interleukin 35 (IL-35) at metastatic sites which activates the JAK2/STAT6/GATA3 signaling pathway to reverse epithelial-mesenchymal transition (EMT) in cancer cells to a mesenchymal- epithelial transition (MET) phenotype, therefore enabling metastatic colonization (Lee et al., 2018). These findings propose TAM-secreted IL-35 as a potential target to intercept metastasis in cancer patients (Lee et al., 2018). Lastly, colony-stimulating factor 1 (CSF-1) and its receptor CSF-1R regulate survival and differentiation of phagocytic myeloid cells and macrophages in particular (Cannarile et al., 2017). *In vitro, in vivo*, and clinical blockade of macrophage CSF-1R with a monoclonal antibody (RG7155) strongly reduced TAM migration and infiltration into the tumor site and the CD8+/CD4+ T cell ratio (Ries et al., 2014). Moreover, targeting of TAMs with a selective CSF-1R inhibitor (AZD7507) in a genetic PDAC mouse model dramatically reduced tumor growth, enhanced T cell immune response and increased mouse survival in a difficult-to-treat model (Candido et al., 2018). In summary, these recent publications support that TAMs are feasible and tenable targets in cancer.

### Molecular Targets in MDSCs

Several MDSC mediated pathways have recently been published highlighting these myeloid cells as novel targets. First, key transmembrane receptor tyrosine kinases (TAM RTK) regulate the innate immune system by dampening inflammatory responses including TYRO3, AXL, MERTK (Graham et al., 2014). TAM RTK are stimulated by protein ligands such as GAS6 and PROTEIN S (Geng et al., 2017). MDSCs were found to dramatically up-regulate TAM RTK and their ligands (Holtzhausen et al., 2019). Genetic or pharmacological inhibition of TAM RTK diminished MDSC suppressive capacity, slowed tumor growth, increased CD8+ T cell infiltration to the tumor site, and augmented anti-PD-1 therapy effect in a melanoma syngeneic mouse model (Holtzhausen et al., 2019). Thus, TAM RTK represents a novel MDSC target in melanoma and potentially other cancers. Second, like TAMs, lipid metabolism is also critical to phenotype. PMN-MDSCs upregulate fatty acid transport protein 2 (FATP2). Genetic deletion or pharmacologic inhibition of FATP2 abrogated the suppressive activity of PMN-MDSCs and delayed tumor growth in multiple syngeneic mouse models. Additionally, FATP2 inhibition blocked tumor growth in combination with immune checkpoint inhibitors, thus highlighting FATP2 as an attractive novel target of MDSCs (Veglia et al., 2019). Makowski lab has demonstrated that FATP1 was critical to the anti-inflammatory M2-like macrophage phenotype, but the role of myeloid FATP1 in the TME is unknown (Johnson et al., 2016; Zhao et al., 2017). Third, AMP-activated protein kinase alpha 1 (AMPKα) is another novel MDSC target which has been previously well documented in other immune populations (Rao et al., 2015, 2016; Zhu et al., 2015). AMPKα upregulation in MDSCs was induced by tumor-secreted granulocyte-monocyte colony-stimulating factor (GM-CSF) in a STAT5-dependent manner. Genetic or pharmacological ablation of AMPKα in several syngeneic mouse models of different cancer types inhibited the immunosuppressive potential of MDSCs, induced CD8+ T cell infiltration into tumor sites, and improved efficacy of immunotherapy (Trillo-Tinoco et al., 2019). These findings support the therapeutic use of AMPK-inhibitors to overcome various immune cell including MDSC-induced immune suppression in cancer (Trillo-Tinoco et al., 2019). Fourth, another novel MDSC target, tumor necrosis factor-alpha-induced protein 8-like 2 (TIPE2), was recently described. TIPE2 expression on MDSCs is induced by reactive oxygen species (ROS) produced by tumor cells. Genetic deletion of TIPE2 or pharmacological inhibition of ROS markedly reduced tumor growth in mice. These findings indicate that TIPE2 plays a critical role in the functional polarization of MDSCs and represents a novel therapeutic target in cancer immunotherapy (Yan et al., 2019). Fifth, therapeutic liver-X nuclear receptor (LXR) agonism reduced MDSC abundance in several syngeneic mouse models and in patients in phase I clinical trials. LXR agonism depletion of MDSC was mediated by its transcriptional target ApoE, where LXR/ApoE activation therapy enhanced T cell activation and potentiated a robust antitumor immune response. Additionally, LXR agonism improved immune checkpoint inhibitor therapies in several preclinical mouse models, thus suggesting LXR agonism as a novel therapeutic approach to deplete MDSCs in cancer patients (Tavazoie et al., 2018). Finally, in contrast to MDSC depletion, a recent study shows that p53 activation induced MDSC differentiation to cross-presenting DCs. Pharmacological activation of p53 induced MDSC differentiation to Ly6C+ CD103 DCs, which are essential to potentiate a CD8+ T cell antitumor immune response. Mice with a targeted deletion of p53 in myeloid cells selectively lost the Ly6C+ CD103+ DC population and failed to respond to multiple forms of immunotherapy. In contrast, p53 agonism markedly enhanced efficacy and duration of response during immunotherapy. Taken together, these recent findings propose a novel therapeutic approach to induce MDSC differentiation to antigen-presenting cells rather than causing their depletion (Sharma et al., 2018).

## Pharmacologic Strategies to Target Myeloid Cells in Cancer

**FIGURE 2:**
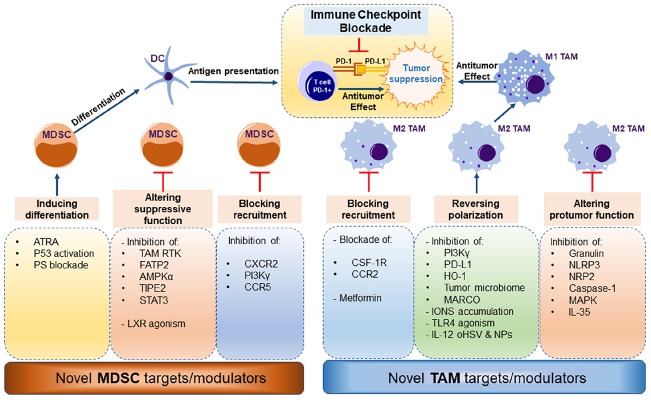
Novel strategies to target myeloid cells in cancer. MDSCs can be differentiated to DCs with ATRA (All Trans Retinoic Acid), p53 activators, or phosphatidylserine blockade. Novel molecular targets altering MDCS suppressive function include inhibition of targets: TAM RTK (TAM Receptor Tyrosine Kinase), FATP2 (Fatty Acid Transport Protein 2), AMPK (5′ AMP-activated protein kinase), TIPE2 (TNF-α-induced protein 8-like 2), STAT3 (Signal Transducer and Activator of Transcription 3) and in contrast, agonism of LXR (Liver X Receptor). Recruitment of MDSCs to the tumor site can be achieved by inhibiting: CXCR2 (C-X-C Motif Chemokine Receptor 2), Pi3kgamma (Phosphoinositide 3-kinase gamma), CCR5 (C-C chemokine receptor type 5). TAMs can be targeted by blocking their recruitment to the tumor site by blocking CSF-1R (Colony Stimulating Factor 1 Receptor) or CCR2, or metformin treatment. Reversing TAM polarization from M2-like to M1-like phenotype can be achieved by inhibition of: Pi3Kgamma, PD-L1 (Protein Death Ligand 1), HO-1 (Heme Oxygenase 1), microbiome ablation, MARCO (macrophage receptor with collagenous structure) blockade, iron accumulation, TLR4 (Toll-like Receptor 4) activation, and IL-12 (Interleukin 12) nanoparticles. Finally, altering TAM function is achieved by inhibition of granulin, NLRP3 (NOD-, LRR- and pyrin domain-containing protein 3), NRP2 (Neuropilin 2), Caspase-1, MAPK (mitogen-activated protein kinase) and IL-35. Depletion of either MDSC and/or M2-like TAMs relieves the immune suppressive burden on T cells and the combination of ICB antibodies further prevents immune evasion by cancer cells leading to tumor suppression.

Recent advances revealing the role of myeloid cells in cancer are drawing more interest in developing effective therapies that would improve prognosis of patients with different cancer types. Recent strategies for targeting the myeloid cell compartment in cancer consist of monotherapies, combination therapies, and/or targeted therapies such as nanoparticles. Recent ongoing and completed clinical trials specifically targeting TAMs and MDSCs in cancer are summarized in Table 1. Here we summarize novel preclinical approaches targeting myeloid cells in cancer (Figure 2).

**TABLE 1:** Current clinical trials targeting Myeloid Derived Suppressor Cells and Tumor Associated Macrophages.

**Compound (target)**	**Clinical phase (status)**	**Tumor type**	**Combination partner(s)**	**ClinicalTrial.gov references**
***MDSCs***				
Ibrutinib (BTK)	Phase I (ongoing)	Solid tumors	Nivolumab	NCT03525925
Tadalafil (PDE5)	NA (completed)	Head and Neck cancer	NA	NCT00843635
RGX-104 (LXR agonism)	Phase I (ongoing)	Solid tumors and lymphoma	Nivolumab/Ipilimumab/Docetaxel/Pembrolizumab, Carboplatin and Pemetrexed	NCT02922764
IPI-549 (Pi3kγ)	Phase II (ongoing)	Breast cancer and renal cell carcinoma	Atezolizumab/nab-paclitaxel/Bevacizumab	NCT03961698
VESANOID (ATRA)	Phase II (ongoing)	Melanoma	Ipilimumab	NCT02403778
Entinostat (HDAC)	Phase I (ongoing)	Breast cancer	Ipilimumab/Nivolumab	NCT02453620
Hydroxychloroquine (autophagy)	Phase I/II (ongoing)	Renal cell carcinoma	IL-2	NCT01550367
Omaveloxolone (NF-κB)	Phase I/II (completed)	Melanoma	*Ipilimumab/Nivolumab*	NCT02259231
beta-glucan (adjuvant)	NA (ongoing)	Non Small Lung cancer	NA	NCT00682032
Capecitabine (thymidylate synthase)	Phase I (ongoing)	Glioblastoma	Bevacizumab	NCT02669173
P53MVA (p53)	Phase II (ongoing)	Ovarian cancer	Pembrolizumab	NCT03113487
***TAMs***				
Pexidartinib (CSF-1R)	Phase I/I (ongoing) (completed)	Sarcoma Glioblastoma Breast cancer Acute myeloid leukemia	Sirolimus Radiotherapy and temozolomide Neoadjuvant chemotherapy NA	NCT02584647 NCT01790503 NCT01042379 NCT01349049
AMG 820 (Anti CSF-1R antibody)	Phase I (completed)	Solid tumors	NA	NCT01444404
LY3022855 (Anti CSF-1R antibody)	Phase I (completed)	Solid tumors Breast/prostate cancer Solid tumors	Durvalumab and Tremelimumab NA NA	NCT02718911 NCT02265536 NCT01346358
Ibrutinib (Bruton kinase)	Phase I (completed)	Pancreatic adenocarcinoma	FOLFIRINOX	NCT02436668
IPI-549 (Pi3kγ)	Phase II (ongoing) Phase I (ongoing)	Breast cancer and renal cell carcinoma Bladder/urothelial cancer Breast/ovarian cancer	Atezolizumab/nab-paclitaxel/Bevacizumab Nivolumab AB928/liposomal doxorubicin/nab-paclitaxel	NCT03961698 NCT03980041 NCT03719326
PF-04136309 (CCR2)	Phase I (completed)	Pancreatic adenocarcinoma	FOLFIRINOX	NCT01413022
Carlumab (Anti-CCL2 antibody)	Phase I (completed)	Solid tumors	Gemcitabine/paclitaxel/carboplatin	NCT01204996
CP-870,893 (CD40 agonist)	Phase I (completed)	Melanoma Solid tumors Pancreatic adenocarcinoma	NA Paclitaxel/carboplatin Gemcitabine	NCT02225002 NCT00607048 NCT01456585
Hu5F9-G4 (Anti-CD47 antibody)	Phase I (completed)	Myeloid leukemia	NA	NCT02678338
BMS-813160 (CCR2)	Phase I/II (ongoing)	Colorectal/pancreatic cancer	Nivolumab/nab-paclitaxel/gemcitabine/5-FU/leucovorin/irinotecan	NCT03184870
MCS110 (Anti-M-CSF antibody)	Phase II (ongoing)	Triple negative breast cancer	Carboplatin/gemcitabine	NCT02435680

*Data were obtained using http://clinicaltrials.gov. BTK, Bruton Tyrosine Kinase; PDE5, phosphodiesterase type 5; LXR, Liver X Receptor; Pi3kγ, Phosphoinositide 3-Kinase gamma; ATRA, All Trans Retinoic Acid; HDAC, Histone deacetylase; NF-κB, nuclear factor kappa-light-chain-enhancer of activated B cells; CSF-1R, Colony Stimulating Factor Receptor 1; CCR2, C-C chemokine receptor type 2; CCL2, C-C chemokine ligand type 2). FOLFIRINOX is a chemotherapy cocktail composed of: folinic acid, fluorouracil, irinotecan, oxaliplatin.*

### Novel Single Agent-Based Potential Therapies

Chemotherapy has been in clinical use since the 1940’s. Paclitaxel is a chemotherapeutic agent isolated from the bark extract of the Pacific Yew Tree in the 1960s. It stabilizes β-tubulin thus blocking mitosis, causing cell cycle G0-phase arrest, and is currently approved by the FDA for the treatment of several cancer types (Wani and Horwitz, 2014; Barbuti and Chen, 2015). Because of the extreme hydrophobicity of paclitaxel, nanoparticle albumin-bound paclitaxel (nab-paclitaxel) has been formulated and approved by the FDA as a first-line treatment of cancer types such as PDAC (Hennenfent and Govindan, 2006). Recent evidence revealed that paclitaxel not only induces cell-cycle arrest, but also promotes antitumor immunity by skewing TAMs toward the M1 phenotype. *In vitro* and *in vivo* tumor models showed that paclitaxel reprogrammed M2-TAMs to the M1-like phenotype in a Toll-like-receptor 4 (TLR4)-dependent manner (Wanderley et al., 2018). In a similar study, nab-paclitaxel was internalized by macrophages via macropinocytosis and induced the M1 phenotype in a TLR4-dependent manner in PDAC *in vitro* and *in vivo* models (Cullis et al., 2017). These data provide a rationale for combination of paclitaxel and immunotherapies as an anticancer treatment approach.

Additionally, a recent study found that the pattern recognition scavenger receptor (MARCO) on TAMs drives immunosuppression. Treatment of breast and colon carcinoma mouse models with an anti-MARCO monoclonal antibody reprogrammed TAMs to a pro-inflammatory phenotype and increased tumor immunogenicity, suggesting that targeting MARCO in TAMs represents a promising mode of cancer treatment (Georgoudaki et al., 2016). Also, all-trans retinoic acid (ATRA) is an active derivation of vitamin A which has an anticancer effect mostly in hematological malignancies (Wansley et al., 2013; Ying et al., 2016). In an osteosarcoma *in vitro* and *in vivo* models, ATRA inhibited osteosarcoma metastasis via inhibiting M2 polarization of TAMs independent of STAT3/6 or C/EBPβ signaling, thus proposing ATRA as an anti-metastatic potential treatment in osteosarcoma patients (Zhou et al., 2017). Another metabolite with potent signaling in myeloid cells is phosphatidylserine. Phosphatidylserine is a phospholipid that contributes to the establishment of an immunosuppressive TME by preventing inflammatory reactions (McDonald et al., 1999). Treatment of prostate tumor-bearing mice with phosphatidylserine-targeting antibody 2aG4 in combination with docetaxel potently suppressed tumor growth, decreased M2 TAM and MDSC populations, and increased M1 macrophage and DC populations in the tumors. Furthermore, 2aG4 repolarized M2 TAMs toward the M1 phenotype, and induced the differentiation of MDSCs into M1 macrophages and DCs *in vitro*. These data suggest that targeting phosphatidylserine could reactivate antitumor immunity in the clinical setting (Yin et al., 2013). Metformin was originally established as the first-line agent for the treatment of type-2 diabetes. However, accumulating data suggest an anticancer effect of metformin in several cancer types (Schuler et al., 2015; Han et al., 2017; Tong et al., 2017; Guo et al., 2019). By using a transgenic adenocarcinoma of the mouse prostate (TRAMP) mouse model, a new study revealed that metformin delayed prostate cancer progression by inhibiting recruitment and infiltration of macrophages to the tumor site. Additionally, metformin inhibited inflammatory macrophage infiltration by downregulating both COX2 and PGE_2_ in tumor cells, suggesting that metformin suppresses prostate cancer by altering tumor TAM infiltration (Liu et al., 2018). Additional pharmacological approaches use phosphodiesterase-5 (PDE-5) inhibitors such as sildenafil to deactivate MDSCs by interfering with Arg1 and iNOS expression (Serafini et al., 2006), N(G)-Nitro-L-Arginine Methyl Ester (L-NAME) which is another compound that inhibits Arg1 activity (Capuano et al., 2009), and N-hydroxy-L-Arginine (NOHA), a potent physiologic inhibitor of Arg1 (Stuehr et al., 1991). In this review, we focused on some recent strategies to inhibit MDSCs in cancer. Additional information regarding other MDSC inhibitors are detailed in another review (Wesolowski et al., 2013). In summary, we suggest several novel targets as a single therapy approach to primarily inhibit immunosuppression by targeting myeloid cell. Although monotherapies targeting myeloid cells in cancer have shown some promising results preclinically and clinically, they still face challenges such as resistance, partial reduction of tumor growth, and the existence of positive crosstalk between myeloid cells and other stromal components which can alter the efficiency of myeloid cell-induced antitumor immunity. It is likely that some of these challenges can be overcome using combination therapies and/or targeted therapies for a greater antitumor effect.

### Potential Combination Therapies Targeting Myeloid Cells in Cancer

In healthy conditions, tumor cells are eliminated by immune surveillance, mainly through T cell infiltration and activation that respond to tumor neoantigens presented by major histocompatibility complex (MHC) (Vesely et al., 2011; Matsushita et al., 2012). However, increased presentation of neoantigens likely leads cancer cells to escape immune surveillance through co-evolution in an immunosuppressive TME (Moon et al., 2014; Khalil et al., 2016). Thus, targeting more than one cellular component of TME in primary and metastatic tumors may provide a solution to immune surveillance evasion by cancer cells to induce a more favorable therapeutic outcome in patients with malignancies. Here, we summarize novel potential combination therapies involving myeloid cells in cancer.

As we have previously mentioned in this review article, targeting PI3Kγ in myeloid cells restored antitumor immunity by switching macrophage polarization toward the proinflammatory phenotype, and induced CD8+ T cell infiltration into the tumor site (Kaneda et al., 2016b). Combination of a PI3Kγ selective inhibitor (IPI-549) with ICB antibodies restored sensitivity of resistant cancers to ICB therapies in preclinical mouse models (De Henau et al., 2016). Likewise, inhibition of MDSCs with IPI-145, a selective inhibitor of PI3Kδ and PI3Kγ isoforms, in combination with anti-PD-L1 induced CD8+ T cell-dependent tumor growth reduction in a head and neck cancer mouse model (Davis et al., 2017). Combination of IL-12-expressing oncolytic herpes simplex virus (oHSV), which selectively replicates in cancer cells, with ICB antibodies (PD-1, CTLA-3, or PD-L1) slightly improved survival of a glioma mouse model. However, triple combination of the IL-12-expressing virus, anti-PD-1, and anti-CTLA-4 cured most mice with an increase of M1 macrophages and T effector to T regulatory ratio into tumors, suggesting that combination macrophage-targeting IL-12-expressing virus and ICB antibodies may have a synergistic curative effect in glioblastoma patients (Saha et al., 2017). As we have previously described in this review, targeting of CSF-1R with a monoclonal antibody (RG7155) potently inhibited TAM recruitment into the tumor site while increasing the anti-tumor CD8+/CD4+ T cell ratio (Ries et al., 2014). A positive effect of CSF-1R blockade plus ICB (anti-PD-1) combination has been reported (Peranzoni et al., 2018). However, a more recent study has identified the mechanism that limited the therapeutic effect of CSF-1R blockade. Carcinoma-associated fibroblasts (CAF) are major recruiter of granulocytes into the tumor site via chemokine secretion (Kumar et al., 2017). CSF-1R blockade induced a profound increase in CAF-mediated MDSC recruitment to the tumor site, thus explaining the mechanism behind CSF-1R therapy limitations. Triple combination of a CSF-1R inhibitor, a CXCR2 antagonist, and anti-PD-1 antibody lead to a significant inhibition of tumor growth in several cancer mouse models. These data propose a novel combination therapy to disrupt the crosstalk between different stromal cell populations for the most efficacious disease outcome in cancer patients (Kumar et al., 2017). In a short communication article, Lorio et al. reported that blocking anti-Bcl-2-Associated athanoGene 3 (BAG3) with an antibody resulted in an increased number of CD8+ T cell infiltration to the tumor site in a PDAC mouse model. Furthermore, combination of anti-BAG3 and anti-PD-1 antibodies further increased CD8+ T cell-mediated antitumor immunity suggesting a novel potential therapeutic approach for the treatment of PDAC (Iorio et al., 2018). Entinostat is an orally bioavailable class I-specific histone deacetylase inhibitor (HDACi) that interrupts immune suppression in the TME (Shen et al., 2012, 2016). Combination of entinostat with anti-PD-1 antibody enhanced the ICB antitumor effect in two syngeneic tumor mouse models by reducing tumor growth and neutralizing both M-MDSC and PMN-MDSC populations (Orillion et al., 2017). Dual targeting of CXCR2+ neutrophils and CCR2+ TAMs increased antitumor immunity by disrupting myeloid recruitment to tumors of a PDAC mouse model and improved response to FOLFIRINOX chemotherapy (including folinic acid, fluorouracil, irinotecan, oxaliplatin). However, targeting of either myeloid subtype (neutrophils or TAMs) resulted in a compensatory response of the other myeloid subset, resulting in disease relapse cite. These data suggest that combination therapies aiming at targeting more than one myeloid subtype in cancer might provide a solution to compensatory mechanisms between stromal cells and may further ameliorate the overall survival of cancer patients (Nywening et al., 2018). In PDAC mouse models, activation of macrophages using a CD40 agonist induced interferon-γ and CCL2 release, which in turn caused macrophages to deplete fibrosis through matrix metalloprotease activity (Beatty et al., 2015; Long et al., 2016). Moreover, combination of CD40 agonist and chemotherapy induced T cell-dependent reduction in tumor growth (Beatty et al., 2015; Long et al., 2016). Combinations involving blockade of leucine-rich repeat-containing G-protein-coupled receptor 4 (Lgr4) in lung cancer (Tan et al., 2018), inhibition of IL-6 in melanoma-bearing mice (Tsukamoto et al., 2018), dietary protein restriction (Orillion et al., 2018), inhibition of casein kinase 2 (Hashimoto et al., 2018), and blockade of receptor-interacting serine/threonine protein kinase 1 (RIP1) in PDAC mouse model (Wang W. et al., 2018) in myeloid cells; and ICB (anti-PD-1, anti-PD-L1, anti-CTLA-4) therapy, synergistically enhanced the antitumor immune response. Taken together, these findings emphasize the importance of targeting myeloid cells in combination with ICB therapies and other therapeutic approaches to enhance the antitumor immune response in cancer patients.

### Targeting Myeloid Cells in Cancer Using Nanoparticles

There has been a growing interest in using nanotechnology for the treatment of cancer in the past few years, which is mainly due to its broad use ranging from drug delivery to diagnosis and imaging (Swartz et al., 2012; Kearney and Mooney, 2013). Nanoparticles are particles of any shape which size ranges between 1 and 100 nm, as defined by the International Union pf Pure and Applied Chemistry (IUPAC) (Shi et al., 2016). The immune system is characterized by its unique specificity in targeting antigens and cancer cells through the innate branch, while its adaptive branch enables long-term activity through memory-driven responses. Thus, manipulating these unique properties of the immune system are desirable, yet come with risks such as immune-related adverse events or “cytokine storm/cytokine release syndrome” (Shimabukuro-Vornhagen et al., 2018). Thus lowering doses and/or targeting specific cells of interest is paramount to ensure patient safety. Nanoparticles are thus excellent candidates to modulate the immune system (Amoozgar and Goldberg, 2015). Here, we briefly summarize recent progress in targeted delivery to specifically myeloid cells in cancer using nanocarriers. Nanoparticle uptake by macrophages is influenced by size, rigidity, shape, surface charge, and composition of the nanoparticle (Tabata and Ikada, 1988; He et al., 2010; Sosale et al., 2015). Also, nanoparticles with either highly positive or highly negative zeta potential, which is defined as the potential difference between dispersion medium and the stationary layer of fluid attached to the particle, are more favorably internalized compared to nanoparticles with neutral or slightly negative charges (Tabata and Ikada, 1988). Several groups have taken advantage of the ability to coat nanoparticles in molecules such as the sugar mannose to target specific myeloid cells. Mannose receptor (CD206) is overexpressed in M2 macrophages and TAMs and represents a suitable target for mannose nanoparticles. Polyethylene glycol (PEG)-sheddable, mannose-modified nanoparticles were developed and efficiently targeted TAMs via mannose-CD206 binding after pH-sensitive PEG dissociated in the acidic TME, while their uptake by normal macrophages was reduced due to efficient PEG shielding at neutral pH (Zhu et al., 2013). Indeed, delivery of silencing molecules (siRNA) to target pro-tumor transcription factors has been undertaken with positive outcomes in an *in vitro* model of ovarian cancer (Ortega et al., 2016). Folic acid liposome nanoparticles were also developed to deliver zoledronic acid to TAMs. Folic acid engaged its receptor folate receptor β (FRβ) which is also overexpressed on TAMs (Hattori et al., 2015). Legumain and transferrin receptors are also overexpressed on TAMs, but all nanoparticle systems that have been developed to target these two receptors are mainly tailored toward targeting cancer cells, but no reports involving TAMs have been communicated yet (Wu et al., 2006; Lin et al., 2013; Ngambenjawong et al., 2017). In another study, IL-12-loaded tumor environment sensitive poly-β-amino ester nanoparticles reeducated TAMs to a macrophage M1 phenotype both *in vitro* and *in vivo* and selectively accumulated in the tumor site while extending IL-12 circulation time (Wang et al., 2017). In summary, we selectively summarized some approaches aiming at targeting myeloid cells in cancer using nanocarriers. Other reviews have extensively detailed other approaches using targeted delivery of myeloid cells in cancer (Amoozgar and Goldberg, 2015; Ngambenjawong et al., 2017; Silva and Al-Jamal, 2017; Singh et al., 2017).

### Toll-Like Receptor Activation in Myeloid Cells

Toll-like receptors (TLR) are transmembrane proteins that induce the activation of inflammatory innate immune responses after binding to microbially derived molecules (Kawai and Akira, 2011; O’Neill et al., 2013). TLR7 and TLR8 agonist R848 potently drove the M1 macrophage phenotype *in vitro*. R848-loaded β-cyclodextrin nanoparticles (CDNP-R848) induced M1 macrophage phenotype, reduced tumor growth, and protected the animals against tumor re-challenge in multiple tumor mouse models. Furthermore, combination of CDNP-R848 and anti-PD-1 antibody improved ICB response (Rodell et al., 2018). In another study, STAT3 small interfering (si)RNA conjugated to cpG oligonucleotide agonist of TLR9 targeted tumor associated myeloid cells by silencing STAT3, thus leading to a potent antitumor immune response in multiple tumor mouse models (Kortylewski et al., 2009) and prostate cancer patients (Hossain et al., 2015). These data demonstrate that activating TLR in myeloid cells using agonists conjugated to therapeutic agents may represent a promising therapeutic approach for patients with different cancer types. Food and Drug Administration (FDA)-approved Toll-like receptor 7 (TLR7) agonist imiquimod is being tested in more than 100 clinical trials as a monotherapy, or in combination with chemotherapy or ICB (Locy et al., 2018). Although imiquimod induces local accumulation and activation of DCs, it may also promote MDSC expansion, which can limit vaccine efficiency (Dang et al., 2012). The mechanism responsible for MDSC activation by some adjuvant therapies is likely due to MDSC’s susceptibility to be triggered by inflammatory signals (Gabrilovich et al., 2012; Fernandez et al., 2014; Kumar et al., 2016). Hence, some TLR agonists therapies have to be combined with agents targeting MDSCs to prevent MDSC-induced immune suppression.

## Concluding Remarks

Before the recent advances in the field of immunotherapy, efforts aimed at targeting cancer were purely one-dimensional by focusing only on cancer cells as a single element in the equation using chemotherapy as early as the 1940’s. With the discovery of TME dynamics and the emergence of immunotherapy, other stromal cell populations are increasingly considered. It is now well established that myeloid cells play a pivotal role in cancer. Their involvement in tumor progression and immune suppression is generating enthusiasm in the cancer research community, especially with the increasing number of novel molecular target discoveries. Although initiatives that aim at targeting myeloid cells in the TME have shown promising results, there still are challenges that need to be resolved. For instance, monotherapies targeting a single myeloid cell phenotype may show promising but limited efficacy. Combination therapies involving different immunotherapeutic approaches show improved anticancer effects in preclinical studies but have not yet lived up to their promises. Perhaps it is essential to discover novel mechanisms involving different stromal components such as the direct and compensatory crosstalk involving CAFs, TAMs, and MDSCs described above (Kumar et al., 2017). In this study, single targeting of TAMs using CSF-1R blockade did not result in a prolonged antitumor immune response. The authors showed that targeting TAMs using CSF-1R blockade triggered a compensation mechanism wherein CAFs recruited more PMN-MDSCs in a CXCR2-depedent manner. Triple combination using CSF-1R, CXCR2, and ICB resulted in an improved antitumor immune response. Thus, discovery of unknown pathways between immune stromal cells may improve cancer treatment by addressing the complexity of stromal interactions in the TME. Moreover, several strategies aiming at achieving an effective combination with ICB are under active investigation (Popovic et al., 2018). The central dogma of these strategies consists of increasing effector T cells in immunologically “cold” tumors which are defined by having low neoantigen burden and a paucity of T cells and DCs (Popovic et al., 2018). One strategy to induce T cells and overcome a “cold” tumor’s TME burden uses vaccines such as the FDA-approved Toll-like receptor 7 (TLR7) agonist imiquimod. As we previously mentioned, imiquimod activates MDSCs and their immunosuppressive capacity thus dampening imiquimod’s antitumor efficacy. Hence, strategies that enhanced DC and T cell antitumor potential while altering MDSC’s suppressive function are likely to be effectively combined with ICB for a maximum therapeutic benefit. Nevertheless, myeloid cells remain a major player that can determine disease outcome in cancer patients because of their exceptional phenotypic plasticity. It is therefore essential to efficiently modulate myeloid cells’ plastic nature for the development of a whole new range of therapeutic strategies against cancer and turn the foes to friends.

## Author Contributions

MC and LM wrote the manuscript. All authors edited the manuscript.

## Conflict of Interest

The authors declare that the research was conducted in the absence of any commercial or financial relationships that could be construed as a potential conflict of interest.
